# Replicability analysis in genome-wide association studies via Cartesian hidden Markov models

**DOI:** 10.1186/s12859-019-2707-7

**Published:** 2019-03-18

**Authors:** Pengfei Wang, Wensheng Zhu

**Affiliations:** Key Laboratory for Applied Statistics of MOE, School of Mathematics and Statistics, Northeast Normal University, 5268 Renmin Street, Changchun, 130024 China

**Keywords:** GWAS, Cartesian hidden Markov model, Replicability analysis

## Abstract

**Background:**

Replicability analysis which aims to detect replicated signals attracts more and more attentions in modern scientific applications. For example, in genome-wide association studies (GWAS), it would be of convincing to detect an association which can be replicated in more than one study. Since the neighboring single nucleotide polymorphisms (SNPs) often exhibit high correlation, it is desirable to exploit the dependency information among adjacent SNPs properly in replicability analysis. In this paper, we propose a novel multiple testing procedure based on the Cartesian hidden Markov model (CHMM), called repLIS procedure, for replicability analysis across two studies, which can characterize the local dependence structure among adjacent SNPs via a four-state Markov chain.

**Results:**

Theoretical results show that the repLIS procedure can control the false discovery rate (FDR) at the nominal level *α* and is shown to be optimal in the sense that it has the smallest false non-discovery rate (FNR) among all *α*-level multiple testing procedures. We carry out simulation studies to compare our repLIS procedure with the existing methods, including the Benjamini-Hochberg (BH) procedure and the empirical Bayes approach, called repfdr. Finally, we apply our repLIS procedure and repfdr procedure in the replicability analyses of psychiatric disorders data sets collected by Psychiatric Genomics Consortium (PGC) and Wellcome Trust Case Control Consortium (WTCCC). Both the simulation studies and real data analysis show that the repLIS procedure is valid and achieves a higher efficiency compared with its competitors.

**Conclusions:**

In replicability analysis, our repLIS procedure controls the FDR at the pre-specified level *α* and can achieve more efficiency by exploiting the dependency information among adjacent SNPs.

**Electronic supplementary material:**

The online version of this article (10.1186/s12859-019-2707-7) contains supplementary material, which is available to authorized users.

## Background

Since the first publication of genome-wide association studies (GWAS) on age-related macular degeneration in 2005 [1], great progress has been made in the genetic studies of the human complex diseases. As of September 1st, 2016, more than 24,000 SNPs have been identified to be associated with complex diseases or traits [2]. It also has been shown that different diseases or traits usually share the similar genetic mechanisms and are even affected by some of the same genetic variants [3, 4]. This phenomenon is known as “pleiotropy". It is desirable to make an integrative analysis of several GWAS studies to improve the power by leveraging the pleiotropy information.

Meta-analysis is one of the approaches that combines of multiple scientific studies and has been widely used in biomedical research. In GWAS, however, the results obtained from meta-analysis are often in contradiction with those in single studies. For example, Voight et al. [5] reported that some of the type 2 diabetes (T2D) related SNPs detected by meta-analysis were not discovered in single studies. It is more convincing if the result can be replicated in at least one study [6]. To this end, replicability analysis was suggested to detect signals that are discovered in more than one study for GWAS [7, 8]. Instead of examining the association in each single study separately, replicability analysis combines results across different studies and can usually gain additional power in genetic association studies. Moreover, it has been reported that the population stratification may affect the GWAS identifications and lead to a subtle bias [9]. We also hope that some of the identified SNPs in the study of one population can be replicated for the studies of other populations. Fortunately, replicability analysis of multiple GWAS from different populations can avoid this kind of bias in some extent.

So far, only a handful of methods have been proposed for replicability analysis. Benjamini et al. [10] utilized the maximum *p*-value of two studies as the joint *p*-value for each test and then carried out the Benjamini-Hochberg procedure [11] to detect replicated signals across two studies. Bogomolov and Heller [12] focused on replicability analysis for two studies, and proposed an alternative FDR controlling procedure based on *p*-values. In 2014, a statistical approach, named GPA, was proposed by [13], which can extract replicated associations through joint analysis of multiple GWAS data sets and annotation information. Heller and Yekutieli [14] extended the two-group model [15] and suggested a generalized empirical Bayes approach, called repfdr, for discovering replicated signals in GWAS. Heller et al. [16] also presented the R package repfdr that provides a flexible and efficient implementation of the method in Heller and Yekutieli [14]. In fact, replicability analysis is a multiple testing problem which involves testing hundreds of null hypotheses that correspond to SNPs without replicated associations. The traditional multiple testing procedures for replicability analysis essentially involve two steps: ranking the hypotheses based on appropriate multiple testing statistics (such as *p*-values) and then choosing a suitable cutoff along with the rankings to ensure the FDR is controlled at the pre-specified level.

It should be pointed out that all these existing approaches assume that the multiple testing statistics (such as *p*-values) are independent in each study, which is obviously unreasonable in practice. For example, in GWAS, since the adjacent genomic loci tend to co-segregate in meiosis, the disease-associated SNPs are always clustered and locally dependent. Wei and Li [17] pointed out that the efficiency of analysis of large-scale genomic data can be evidently enhanced by exploiting genomic dependency information properly. It also has been shown that ignoring the dependence among the multiple testing statistics will decrease the statistical accuracy and testing efficiency in multiple testing [18–20]. Hence a reasonable multiple testing statistic for a given SNP should depend on data from neighboring SNPs in replicability analysis and it is worthy of developing a multiple testing procedure that can take into account the dependency information among adjacent SNPs for each study in replicability analysis.

Recently, the hidden Markov model (HMM) has been successfully applied to large-scale multiple testing under dependence [20]. Since the Markov chain is an effective tool for modelling the clustered and locally dependent structure, it has been successfully applid in GWAS [21–23]. Inspired by their works, we utilize the Cartesian hidden Markov model (CHMM) to characterize the dependence among adjacent SNPs for each study in replicability analysis. Based on CHMM, we develop a novel multiple testing procedure which is referred to as replicated local index of significance (repLIS) for replicability analysis across two studies. The statistics involved in repLIS can be calculated highly effectively by using the forward-backward algorithm. Simulation studies show that our repLIS procedure can control the FDR at the nominal level and enjoys a higher efficiency compared with its competitors. We also successfully apply our repLIS procedure in replicability analyses of psychiatric disorders data sets collected by Psychiatric Genomics Consortium (PGC) and Wellcome Trust Case Control Consortium (WTCCC).

## Results

### Application of detecting the pleiotropy effect

So far, accumulating evidence suggests that many different diseases or traits share the similar genetic architectures and are usually affected by some of the same genetic variants [3, 4]. This phenomenon is referred to as “pleiotropy". It is meaningful to jointly analyze several GWAS data sets to detect the SNPs with pleiotropy information. The cross-disorder group of Psychiatric Genomics Consortium (PGC) is aim to investigate the genetic associations between five psychiatric disorders, including attention deficit-hyperactivity disorder (ADHD), autism spectrum disorder (ASD), bipolar disorder (BD), major depressive disorder (MDD), and schizophrenia (SCZ) [24, 25]. It has been shown that there exists the pleiotropy effect between BD and SCZ [13, 26]. We apply our proposed repLIS procedure to detect the SNPs with pleiotropy effect between BD and SCZ in the data sets collected by the PGC. The *p*-values are available for 2,427,220 SNPs in BD and 1,252,901 SNPs in SCZ, in which 1,064,235 SNPs are used both in BD and SCZ. In this study, we aim to detect the SNPs with pleiotropy effect between BD and SCZ.

**Fig. 1:**
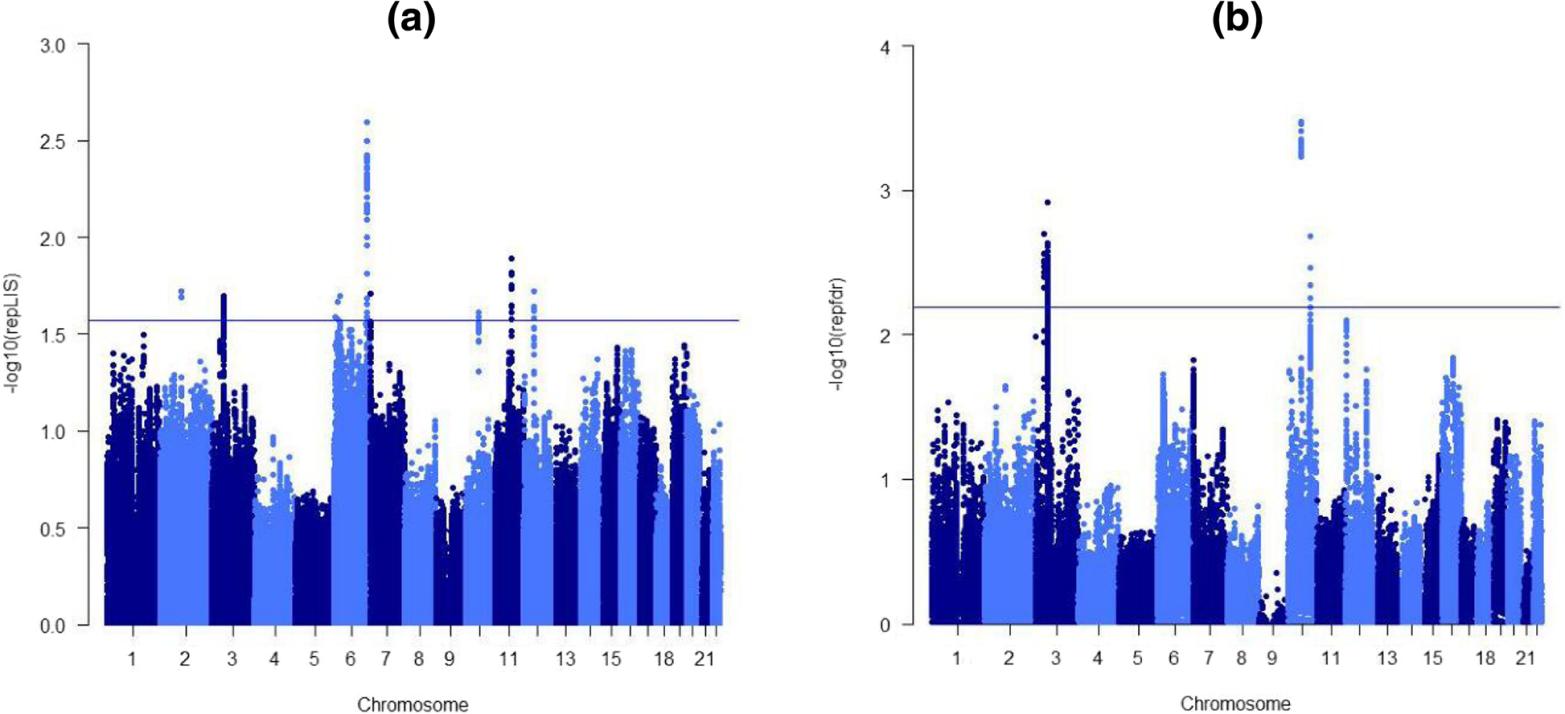
The Manhattan plots for repLIS procedure and repfdr procedure. The horizontal line for each panel is drawn such that there are 100 SNPs with the values of $-\log _{10}\left (\widehat {repLIS}\right)$ or $-\log _{10}\left (\widehat {repfdr}\right)$ above the line. **a** The SNPs above the horizontal line concentrate on chromosome 3, 6, 10 and 11 in the Manhattan plots for repLIS procedure. **b** The SNPs above the horizontal line concentrate on chromosome 3 and 10 in the Manhattan plots for repfdr procedure

Since both repfdr and our repLIS procedure are based on *z*-values, we first calculate the *z*-values transformed by the corresponding *p*-values. In order to avoid the situation that the *z*-value is infinity, we set the *p*-values to be 0.99 if they are recorded to be 1 in the data sets. We compare the results given by repfdr and repLIS for detecting the SNPs with pleiotropy effect. Wei et al. [21] suggested that combining the testing results from several chromosomes is more efficient. Hence we apply the repLIS procedure to calculate the repLIS statistics on each chromosome separately, while the ranking of repLIS statistics is based on all the chromosomes of interest. The Manhattan plots are shown in Fig. 1, and the horizontal line for each panel is drawn such that there are 100 SNPs with the values of $-\log _{10}\left (\widehat {repLIS}\right)$ or $-\log _{10}\left (\widehat {repfdr}\right)$ above the line. In Fig. 1, we can see from panel (b) that the SNPs above the horizontal line concentrate on chromosome 3 and chromosome 10. This indicates that the SNPs identified by repfdr procedure with strong pleiotropy effect are located on chromosomes 3 and 10. Indeed, most of the Top 100 SNPs discovered by repfdr are clustered in the genes IHIH1, IHIH3, GNL3, PBRM1, NEK4, GLT8D1 (on chromosome 3) and ANK3 (on chromosome 10). In addition to these genes identified by repfdr procedure, our repLIS procedure further discoverd genes SYNE1 on chromosome 6 and TENM4 on chromosome 11 with strong pleiotropy effect between BD and SCZ. The findings here support several genetic associations to genes for BD and/or SCZ. For instance, the gene SYNE1 provides instructions for making a protein called Syne-1 which is especially critical in the brain and plays a role in the maintenance of the part of the brain that coordinates movement. It has been shown that SYNE1 is one of the implicated genes in the etiology of BD [25]. Another gene TENM4 (also named ODZ4) has been identified to be co-expressed with miR-708. It has been reported that a single variant located near the miR-708 may have a role in susceptibility to BD and SCZ [27].

### Application of discovering the replicated association

Bipolar disorder (BD) is a manic depressive illness that causes periods of depression and periods of elevated mood. In this section, we further apply our repLIS procedure to the replicability analysis of BD data sets from PGC and Wellcome Trust Case Control Consortium (WTCCC). The data sets collected by WTCCC contain 1998 cases and 3004 controls, among which there are 1504 control samples from the 1958 Birth Cohort (58C) and the other control samples from UK Blood Service (UKBS).

We first conduct a series of procedures for quality control on WTCCC data sets. We eliminate 130 samples from the BD cohort, 24 samples from the 58C cohort and 42 samples from the UKBS cohort owing to the high missing rate, overall heterozygosity, and non-European ancestry. In addition, we remove the SNPs in accordance with the exclusion list provided by WTCCC and exclude the SNPs with minor allele frequency less than 0.05. We fit the logistic regression model for each SNP and obtain the *p*-value of testing for the association between the SNP and the disease of interest. Taking the intersection of SNPs in PGC and WTCCC yields to 361,665 SNPs that are available for replicability analysis.

Since it is unfeasible to validate the true FDR level in real data analysis, we choose an alternative measure, the efficiency of ranking replicated signals, for comparisons. Consortium et al. [28] have identified fourteen BD-susceptibility SNPs that are showing strong or moderate evidence of associations with BD, among which eleven SNPs are simultaneously identified by [29]. We focused on these fourteen SNPs and treated them as relevant SNPs. The performance of replicability analysis procedure is assessed by the ranks of these fourteen relevant SNPs as well as the number of relevant SNPs that are selected by top *k* significant SNPs. Table 1 presents the results of repLIS and repfdr in identifying the relevant SNPs when top *k*=500. repLIS identifies eight of the fourteen relevant SNPs, whereas repfdr only identifies five of those SNPs. Four relevant SNPs (rs7570682; rs1375144; rs2953145; rs10982256) are identified by repLIS only, whereas one SNP (rs3761218) is identified by repfdr only. We can observe that there is a significant improvement of rankings for most of these SNPs with replicated associations when conducting repLIS procedure. For instance, rs420259 that is reported to have a strong association with BD [28] ranks 255th by repfdr procedure and 115th by repLIS procedure.

**Table 1:** Results of repfdr and repLIS procedure when top *k*=500

SNP ID	Chr	repfdr ranks	repLIS ranks	repfdr values	repLIS values
rs7570682	2	−	**35**	1	3.7e-2
rs1375144	2	−	**24**	1	3.1e-2
rs2953145	2	−	**25**	1	3.2e-2
rs4276227^*s*^	3	105	**64**	6.4e-3	4.5e-2
rs683395^*s*^	3	99	**51**	6.1e-3	4.3e-2
rs10982256	9	−	**305**	1	7.9e-2
rs1344484	16	49	**15**	1.9e-3	2.3e-2
rs420259	16	255	**115**	1.5e-2	5.4e-2
rs3761218	20	233	−	1.4e-2	9.9e-1

^*s*^ The SNPs that are only identified by [28] and others are simultaneously identified by [29]. ’ −’ denotes a relevant SNP non-identified by the corresponding procedure. There is a significant improvement of rankings for most of these SNPs with replicated associations when conducting repLIS procedure

**Fig. 2:**
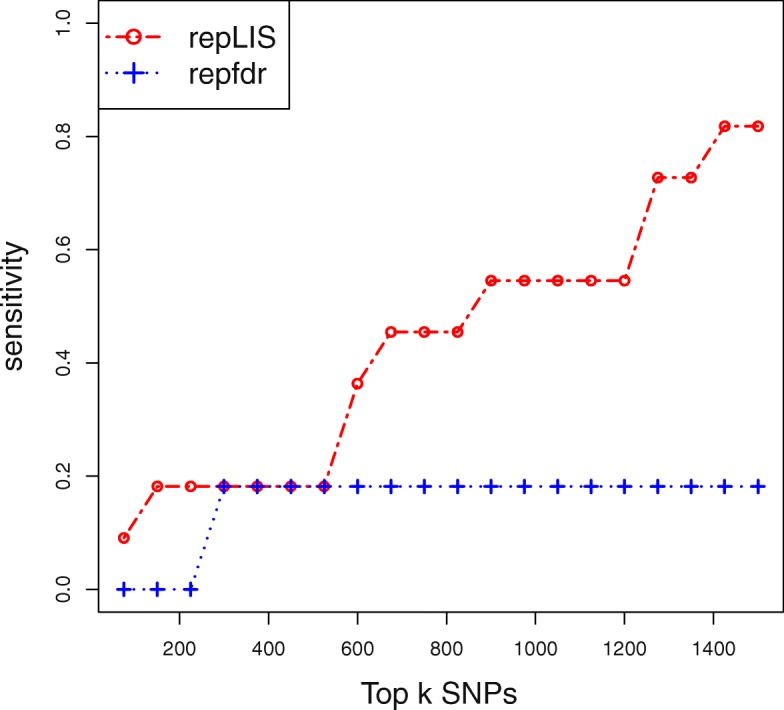
The sensitivity curves yielded by repLIS and repfdr in real data analysis. The results are almost coincide with those in Simulation II

To further illustrate the superiority of repLIS is achieved by leveraging information from adjacent SNPs via a Markov chain, we focused on the adjacent SNPs of rs420259, and selected the five adjacent SNPs on each side of rs420259 as relevant SNPs. We plotted the sensitivity curve in Fig. 2 as described in Simulation II, and obtained very similar results.

## Discussion

In this paper, we propose a novel multiple testing procedure, called repLIS procedure, for replicability analysis across two studies. The repLIS procedure can characterize the local dependence structure among adjacent SNPs via a four-state Markov chain. Based on the CHMM, the multiple testing statistics (repLIS statistics) can be calculated efficiently by using the forward-backward algorithm. When the parameters of CHMM are known, the theoretical results showed that our repLIS procedure is valid and optimal in the sense that repLIS procedure can control the FDR at the pre-specified level *α* and has the smallest FNR among all *α*-level multiple testing procedures. In reality, the parameters of CHMM are usually unknown and hence we further provided the detailed EM algorithm to estimate the parameters of CHMM.

Both the simulation studies and real data analysis exhibit that the repLIS procedure is valid and more efficient by employing the dependency information among adjacent SNPs. Some of the SNPs identified by repLIS have been verified by other researchers. For example, a large number of literatures confirm that rs420259 is really relevant to BD [29–31]. However, some of the other SNPs identified by repLIS have not been verified in previous research (e.g., rs206731), and further experiments need to be conducted to verify the research findings.

The repLIS procedure is implemented by using the R code. We give a brief description of the source code in Additional file 1, and all core code of repLIS procedure are available on GitHub (https://github.com/wpf19890429/large-scale-multiple-testing-via-CHMM).

## Conclusions

Our repLIS procedure can also be extended in several ways. First, it might be a strong assumption that the transition probability (1) is invariant across the whole two studies. It would be of interest to generalize our repLIS from a homogeneous Markov chain to a nonhomogeneous Markov chain or even a Markov random field. Second, the EM algorithm for estimating the parameters of CHMM is a heuristic algorithm and may lead to a local optimum in some situations. The Markov Chain Monte Carlo (MCMC) algorithm which are not relying on the starting point may give rise to a bright way for estimating these parameters. Finally, although this paper considered the repLIS procedure for replicability analysis across two studies, extensions to more than two studies are straightforward by utilizing a multi-dimensional Markov chain to describe the local dependence structure. However, a new issue will arise in multiple testing, since the computation is intractable when the dimension is high. It is desirable to develop a procedure that can handle replicability analysis with a multitude of studies.

## Methods

### Replicability analysis in the framework of multiple testing

In order to express the problem explicitly, we first make a brief description of the framework for replicability analysis across two studies in GWAS. Suppose there are *m* SNPs to be investigated in each study. For the *i*th study (*i*=1,2), let $\left \{H_{i,j}\right \}^{m}_{j=1}$ be the underlying states of the hypotheses, where *H*_*i*,*j*_=1 indicates that the *j*th SNP is associated with the phenotype of interest and *H*_*i*,*j*_=0 otherwise. For the *j*th SNP, we are interested in examining the following null hypothesis

$$\mathcal{H}^{0j}_{NR}: \left(H_{1,j},H_{2,j}\right)\in \left\{(0,0),(1,0),(0,1) \right\}, $$

and we call $\mathcal {H}^{0j}_{NR}$ the no replicability null hypothesis showing that the SNP is associated with the phenotype in at most one study. The goal of the replicability analysis in GWAS is to discover as many SNPs that are associated with phenotype in both studies as possible [14]. In this paper, we handle this problem in the framework of multiple testing under dependence since the disease-associated SNPs are always clustered and dependent. Specifically, we aim to develop a multiple testing procedure that can discover the SNPs with replicated associations (i.e. (*H*_1,*j*_,*H*_2,*j*_)=(1,1)) as many as possible, while the FDR is controlled at the pre-specified level. To this end, we define the FDR as follows:

$$\text{FDR}=E\left[\frac{\sum^{m}_{j=1}I_{\left(\left(H_{1,j},H_{2,j}\right)\in \{(0,0),(1,0),(0,1)\}\right)}\delta_{j}}{\sum^{m}_{j=1}\delta_{j}}\right], $$

where *δ*_*j*_=1 indicates that the *j*th SNP is claimed to be associated with the phenotype in both studies and *δ*_*j*_=0 otherwise. Correspondingly, the marginal false discovery rate (mFDR) is defined as:

$$\text{mFDR}=\frac{E\left[\sum^{m}_{j=1}I_{\left(\left(H_{1,j},H_{2,j}\right)\in \{(0,0),(1,0),(0,1)\}\right)}\delta_{j}\right]}{E\left[\sum^{m}_{j=1}\delta_{j}\right]}. $$

Since the mFDR is asymptotically equivalent to the FDR in the sense that $\text {mFDR}=\text {FDR}+O\left (1/\sqrt {m}\right)$ under some mild conditions [32], hereafter, we mainly focus on developing a multiple testing procedure that can control the mFDR at the pre-specified level for replicability analysis.

### The Cartesian hidden Markov model

Let *z*_*i*,*j*_ be the observed *z*-value of the *j*th SNP in the *i*th association study, which can be obtained by using appropriate transformation. Specifically, *z*_*i*,*j*_ can be transformed from *Φ*^−1^(1−*p*_*i*,*j*_), where *Φ*^−1^ is the inverse of the standard normal distribution and *p*_*i*,*j*_ is the *p*-value of the *j*th SNP in the *i*th association study, for *i*=1,2, and *j*=1,…,*m*.

The Markov chain, which is an effective tool for modelling the clustered and locally dependent structure among disease-assocaited SNPs, has been widely used in the literatures [21, 22]. We assume that $\left \{\left (H_{1,j},H_{2,j}\right)\right \}^{m}_{j=1}$ is a four-state stationary, irreducible and aperiodic Markov chain with the transition probability

1 $$ A_{uv}=P\left(\left(H_{1,j+1},H_{2,j+1}\right)=v|\left(H_{1,j},H_{2,j}\right)=u \right),  $$

where *u*,*v*∈{(0,0),(1,0),(0,1),(1,1)}. We further assume that the observed *z*-values $\left \{\left (z_{1,j},z_{2,j}\right)\right \}^{m}_{j=1}$ are conditionally independent given the hypotheses states $\left \{\left (H_{1,j},H_{2,j}\right)\right \}^{m}_{j=1}$, namely,

2 $$ {{\begin{aligned} P\left(\left\{\left(z_{1,j},z_{2,j}\right)\right\}^{m}_{j=1} | \left\{\left(H_{1,j},H_{2,j}\right)\right\}^{m}_{j=1} \right) =\prod\limits^{m}_{j=1}P\left(z_{1,j}|H_{1,j}\right)\prod\limits^{m}_{j=1}P\left(z_{2,j}|H_{2,j}\right). \end{aligned}}}  $$

**Fig. 3:**
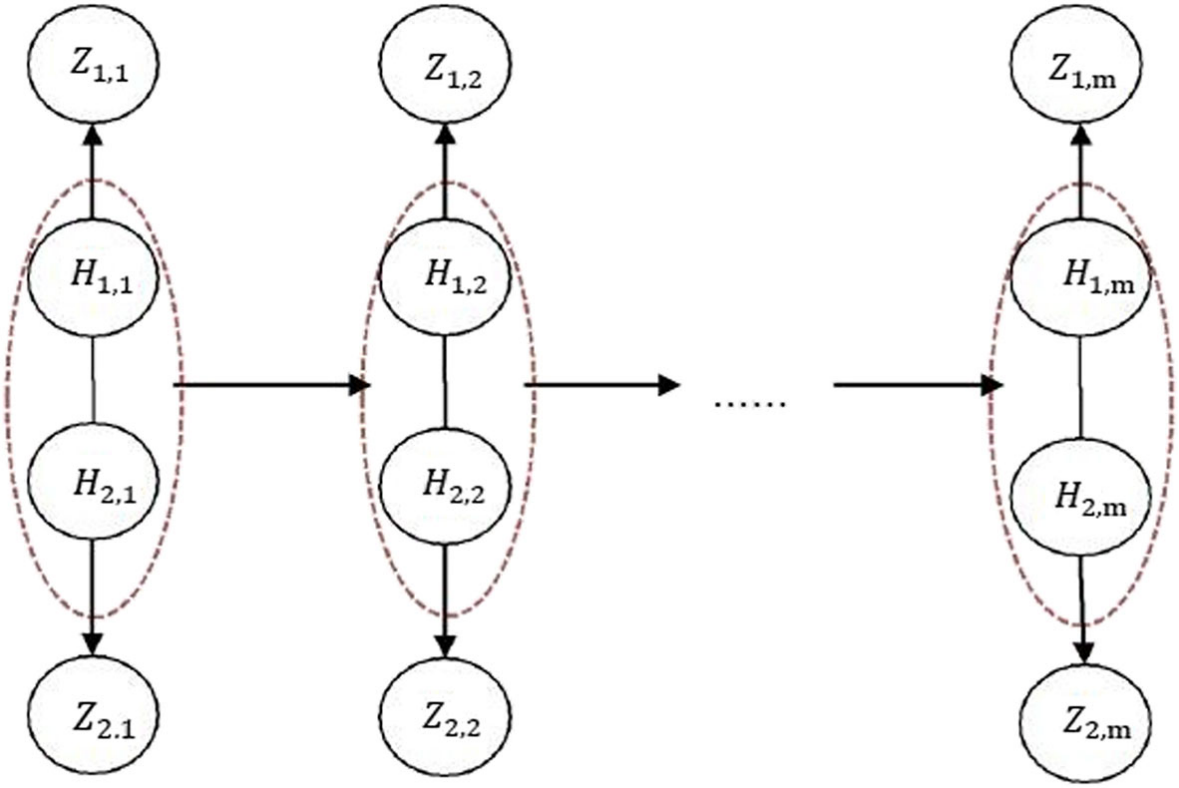
Graphical representation of the CHMM

The Markov chain $\left \{\left (H_{1,j},H_{2,j}\right)\right \}^{m}_{j=1}$ with the dependence model (2) is called Cartesian hidden Markov model (CHMM) [33]. The structure of the CHMM can be intuitively understood with a graphical model as follows in Fig. 3.

Following [20–22], we suppose that the corresponding random variable *Z*_*i*,*j*_ follows the two-component mixture model:

3 $$ Z_{i,j}|H_{i,j}\sim \left(1-H_{i,j}\right)f_{i0}+H_{i,j}f_{i1},  $$

where *f*_*i*0_ and *f*_*i*1_ are the conditional probability densities of *Z*_*i*,*j*_ given *H*_*i*,*j*_=0 and *H*_*i*,*j*_=1, respectively. In practice, we usually assume that *f*_10_ and *f*_20_ are the densities of the standard normal distribution *N*(0,1), and *f*_11_ and *f*_21_ are the densities of the normal distributions $N\left (\mu _{1},\sigma ^{2}_{1}\right)$ and $N\left (\mu _{2},\sigma ^{2}_{2}\right)$, respectively.

Let *π*=(*π*_00_,*π*_10_,*π*_01_,*π*_11_) be the initial distribution of the four-state Markov chain, where *π*_*st*_=*P*((*H*_1,1_,*H*_2,1_)=(*s*,*t*)), for *s*,*t*=0,1. For convenience, let $\vartheta =(\pi,\mathcal {A},\mathcal {F})$ denote the parameters of the CHMM, where $\mathcal {A}=\left \{A_{uv}\right \}_{4\times 4}$ with *u*,*v*∈{(0,0),(1,0),(0,1),(1,1)} and $\mathcal {F}=\left (f_{10},f_{11},f_{20},f_{21}\right)$.

### The repLIS procedure for replicability analysis

In this section, we develop the multiple testing procedure for replicability analysis by studying the connection between the multiple testing and weighted classification problems. Consider the loss function of the weighted classification problem with respect to replicability analysis as

$$ {\begin{aligned} L_{\lambda} \left(\left\{H_{1,j}\right\}^{m}_{j=1},\left\{H_{2,j}\right\}^{m}_{j=1},\left\{\delta_{j}\right\}^{m}_{j=1} \right) & =\frac{1}{m}\sum\limits^{m}_{j=1}\left\{\lambda\left[\left(1-H_{1,j}\right)\left(1-H_{2,j}\right)\right.\right.\\ & \quad\left.+ H_{1,j}\left(1-H_{2,j}\right)+\left(1-H_{1,j}\right)H_{2,j}\right]\delta_{j} \\ &\quad+ \left. H_{1,j}H_{2,j}(1-\delta_{j})\right\}, \end{aligned}} $$

where *λ* is the relative cost of false positive to false negative, and *δ*_*j*_ was defined in the above section and we call (*δ*_1_,…,*δ*_*m*_)∈{0,1}^*m*^ the classification rule for replicability analysis here. By some simple derivations, the optimal classification rule, which minimizes the expectation of the loss function, is obtained as

4 $$ \delta_{j}\left(\Lambda_{j},1/\lambda\right)=I_{\left(\Lambda_{j}<1/\lambda\right)}, ~~ \text{for} ~ j=1, \dots, m  $$

where

$$\Lambda_{j}=\frac{P\left(\mathcal{H}^{0j}_{NR} \text{~is ~true~} \big |\{z_{1,i}\}^{m}_{i=1},\{z_{2,i}\}^{m}_{i=1} \right)}{1-P\left(\mathcal{H}^{0j}_{NR} \text{~is ~true~} \big | \{z_{1,i}\}^{m}_{i=1},\{z_{2,i}\}^{m}_{i=1} \right)} $$

is called the optimal classification statistic in the weighted classification problem, and *I*_(·)_ is an indicator function.

Following the work of [34], it is not difficult to show that the optimal classification statistic is also optimal for replicability analysis in the sense that the multiple testing procedure based on the optimal classification statistics with a suitable cutoff can control the mFDR at the pre-specified level *α* and has the smallest mFNR among all *α*-level multiple testing procedures. Since *Λ*_*j*_ is increasing with $P\left (\mathcal {H}^{0j}_{NR} \text {~is ~true~} |\left \{z_{1,i}\right \}^{m}_{i=1},\left \{z_{2,i}\right \}^{m}_{i=1}\right)$, we can also define the optimal multiple testing statistic for replicability analysis as

5 $$ {{\begin{aligned} \text{repLIS}_{j}=P \left(\mathcal{H}^{0j}_{NR} \text{~is ~true~} \left|\{z_{1,i}\}^{m}_{i=1},\left\{z_{2,i}\right\}\right.^{m}_{i=1} \right), ~~ \text{for}~ j=1, \dots, m. \end{aligned}}}  $$

Denote by repLIS_(1)_, repLIS_(2)_,…,repLIS_(*m*)_ the ordered repLIS values and $\mathcal {H}^{0(1)}_{NR}$, $\mathcal {H}^{0(2)}_{NR}, \dots, \mathcal {H}^{0(m)}_{NR}$ the corresponding no replicability null hypotheses. The repLIS procedure for replicability analysis is:

6 $$ {{\begin{aligned} \text{{let}~} l=\max \left\{t: \frac{1}{t}\sum\limits^{t}_{j=1} \text{repLIS}_{(j)} \leq\alpha \right\}; ~\text{then~ reject~all~} \mathcal{H}^{0(j)}_{NR}, j=1, \dots, l. \end{aligned}}}  $$

It is necessary to note that, to focus on the main ideas, we restrict attention to repLIS in testing two GWAS studies. Extending repLIS to multiple GWAS studies (≥3) is formally straightforward, but requires additional computations.

The following theorem shows that repLIS procedure is asymptotically optimal. The proof of the theorem is outlined in Additional file 2.

#### **Theorem 1**

Consider the Cartesian hidden Markov model (1)-(2) and define the testing statistics $\text {repLIS}_{j}=P\left (\left (H_{1,j},H_{2,j}\right)\in \{(0,0),(1,0),(0,1)\} |\{z_{1,i}\}^{m}_{i=1},\left \{z_{2,i}\right \}^{m}_{i=1}\right)$ for *j*=1,…,*m*. Let repLIS_(1)_, repLIS_(2)_,…,repLIS_(*m*)_ be the ordered repLIS values and $\mathcal {H}^{0(1)}_{NR}$, $\mathcal {H}^{0(2)}_{NR}, \dots, \mathcal {H}^{0(m)}_{NR}$ be the corresponding no replicability null hypotheses. Then the repLIS procedure (6) controls FDR at *α*. Moreover, the FNR yielded by repLIS procedure is *β*^∗^+*o*(1), where *β*^∗^ is the smallest FNR level among all *α*-level FDR multiple testing procedures.

### The forward-backward algorithm for computing repLIS

When the parameters of CHMM are known, repLIS statistics can be calculated by utilizing the forward-backward algorithm. Specifically, the repLIS statistic for the *j*th SNP can be expressed as:

$$\text{repLIS}_{j}=1-\frac{\alpha_{j}(1,1)\beta_{j}(1,1)}{\sum^{1}_{p=0}\sum^{1}_{q=0}\alpha_{j}(p,q)\beta_{j}(p,q)}, $$

where the forward variable $\alpha _{j}(p,q)=P\left ({\vphantom {\left \{z_{1,i}\right \}^{j}_{i=1},\left \{z_{2,i}\right \}^{j}_{i=1}}}\left (H_{1,j},H_{2,j}\right)=\right. \left.(p,q),\left \{z_{1,i}\right \}^{j}_{i=1},\left \{z_{2,i}\right \}^{j}_{i=1}\right)$ and the backward variable $\beta _{j}(p,q)=P\left (\left \{z_{1,i}\right \}^{m}_{i=j+1},\left \{z_{2,i}\right \}^{m}_{i=j+1}|\left (H_{1,j},H_{2,j}\right)=(p,q)\right)$ can be calculated by using the following recursive formulas:

$$\alpha_{j+1}(p,q)=\sum^{1}_{s=0}\sum^{1}_{t=0}\alpha_{j}(s,t)f_{1p}\left(z_{1,j+1}\right)f_{2q}\left(z_{2,j+1}\right)A_{(s,t)(p,q)}, $$

$$\beta_{j}(p,q)=\sum^{1}_{s=0}\sum^{1}_{t=0}\beta_{j+1}(s,t)f_{1s}\left(z_{1,j+1}\right)f_{2t}\left(z_{2,j+1}\right)A_{(p,q)(s,t)}. $$

### The EM algorithm for estimating the parameters of CHMM

In reality, the parameters *𝜗* of the CHMM are not usually known. We use the plug-in $\widehat {\text {repLIS}}$ yielded by utilizing the maximum likelihood estimates to replace the true parameters for replicated analysis. In this section, we provide details of the EM algorithm for estimating the parameters of CHMM. For simplicity, let $\sum \limits _{H_{1,*};H_{2,*}}=\sum \limits _{H_{1,1},H_{1,2},\dots,H_{1,m}} \sum \limits _{H_{2,1},H_{2,2},\dots,H_{2,m}}$, $\mathcal {Z}=\left (\left \{z_{1,j}\right \}^{m}_{j=1},\left \{z_{2,j}\right \}^{m}_{j=1}\right)$ and $\mathcal {H}=\left (\left \{H_{1,j}\right \}^{m}_{j=1},\left \{H_{2,j}\right \}^{m}_{j=1}\right)$.

The full likelihood can be expressed as:

$$\begin{array}{@{}rcl@{}} L(\vartheta;\mathcal{Z},\mathcal{H})&\,=\,&P_{\vartheta}\left(\left\{z_{1,j}\right\}^{m}_{j=1},\!\left\{z_{2,j}\right\}^{m}_{j=1},\!\left\{H_{1,j}\right\}^{m}_{j=1},\!\left\{H_{2,j}\right\}^{m}_{j=1}\right)\\ &=&P_{\vartheta}\left(H_{1,1},H_{2,1}\right)\prod^{m}_{j=1}f_{1H_{1,j}}\left(z_{1,j}\right)\prod^{m}_{j=1}f_{2H_{2,j}}\left(z_{2,j}\right)\\ &&\times \prod^{m-1}_{j=1}A_{\left(H_{1,j},H_{2,j}\right)\left(H_{1,j+1},H_{2,j+1}\right)}. \end{array} $$

We first initialize the parameters $\vartheta ^{(0)}= \left (\pi ^{(0)},\mathcal {A}^{(0)},\mathcal {F}^{(0)}\right)$. In the E-step of the *t*th iteration, we calculate the following *Q*(*𝜗*,*𝜗*^(*t*)^) function:

$$\begin{array}{@{}rcl@{}} Q\left(\vartheta,\vartheta^{(t)}\right)&=&\sum\limits_{H_{1,*};H_{2,*}}\log P_{\vartheta}(\mathcal{Z},\mathcal{H})P_{\vartheta^{(t)}}(\mathcal{Z},\mathcal{H})\\ &=&\sum\limits_{H_{1,*};H_{2,*}}\log P_{\vartheta}\left(H_{1,1},H_{2,1}\right)P_{\vartheta^{(t)}}(\mathcal{Z},\mathcal{H})\\ &&+\sum\limits_{H_{1,*};H_{2,*}}\left[\sum^{m}_{j=1}\log \left.\left(f_{1,H_{1,j}}\left(z_{1,j}\right)f_{2,H_{2,j}}(z_{2,j}\right)\right)\right]P_{\vartheta^{(t)}}(\mathcal{Z},\mathcal{H})\\ &&+\sum\limits_{H_{1,*};H_{2,*}}\left[\sum^{m-1}_{j=1}\log A_{\left(H_{1,j},H_{2,j}\right)\left(H_{1,j+1},H_{2,j+1}\right)} \right]P_{\vartheta^{(t)}}(\mathcal{Z},\mathcal{H}) \end{array} $$

In the M-step of the *t*th iteration, maximizing *Q*(*𝜗*,*𝜗*^(*t*)^) yields to

$$\vartheta^{(t+1)}=\arg\max\limits_{\vartheta}Q\left(\vartheta,\vartheta^{(t)}\right). $$

Specifically, using the Lagrange multiplier method yields to

$$\begin{array}{@{}rcl@{}} \pi^{(t+1)}_{u} &=& P_{\vartheta^{(t)}}\left(H_{1,1},H_{2,1}\right)=u|\mathcal{Z}), \\ A^{(t+1)}_{uv} &\,=\,& \frac{\sum^{m-1}_{j=1}P_{\vartheta^{(t)}}\left(\left(H_{1,j},H_{2,j}\right)\,=\,u,\left(H_{1,j+1},H_{2,j+1}\right)\,=\,v|\mathcal{Z}\right)} {\sum^{m-1}_{j=1}P_{\vartheta^{(t)}}\left(\left(H_{1,j},H_{2,j}\right)=u|\mathcal{Z}\right)}, \\ \mu^{(t+1)}_{i} &=& \frac{\sum^{m}_{j=1}z_{i,j}P_{\vartheta^{(t)}}\left(H_{i,j}=1|\mathcal{Z}\right)} {\sum^{m}_{j=1}P_{\vartheta^{(t)}}\left(H_{i,j}=1|\mathcal{Z}\right)}, \\ \sigma^{2(t+1)}_{i} &= &\frac{\sum^{m}_{j=1}\left(z_{i,j}-\mu^{(t+1)}_{i}\right)^{2}P_{\vartheta^{(t)}}\left(H_{i,j}=1|\mathcal{Z}\right)} {\sum^{m}_{j=1}P_{\vartheta^{(t)}}\left(H_{i,j}=1|\mathcal{Z}\right)}, \end{array} $$

for *i*=1,2 and *u*,*v*∈{(0,0),(1,0),(0,1),(1,1)}.

## Simulation studies

### Simulation I

In this section, we explore the numerical performance of our novel procedures: the oracle repLIS (repLIS.or) and data-driven repLIS (repLIS) procedures, and two existing multiple testing procedures for replicability analysis in testing two GWAS studies, including the Benjamini-Hochberg procedure (BH) [11] and the repfdr procedure (repfdr) [14]. We also carried out further simulation studies for repLIS in testing three GWAS studies. The detailed simulation results are displayed in Additional file 2 and they are almost coincide with those for testing two GWAS studies. We compare these multiple testing procedures in detecting replicated signals from three aspects. First, we check whether or not the FDR values yielded by different procedures are controlled at the pre-specified level *α*, where *α* is set to be 0.1 and 0.02 in the simulation, and the results for *α*=0.02 are illustrated in Additional file 2. Second, we compare the FNR and the average number of true positives (ATP). In general, a valid procedure (the FDR value is contronlled at the pre-specified level) is efficient if it allows for a small FNR value and a large ATP value. In Simulation I, we consider two scenarios based on whether or not the tests of all the SNPs are independent in each study. Third, we investigate the ranking efficiency of these procedures in Scenario 2 of Simulation I. The simulation results are based on 200 replications in Simulation I and the number of tests (i.e. *m*) in each study is 10000 for all the simulations.

#### Scenario 1: independent tests

**Fig. 4:**
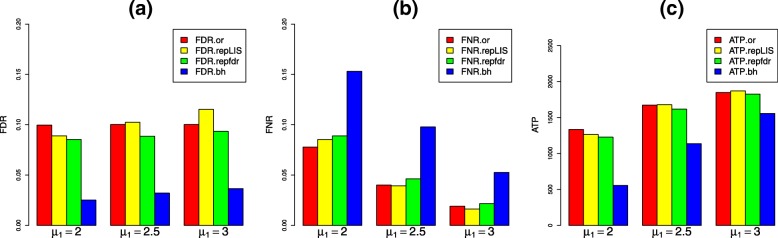
Simulation results in Scenario 1. **a** The FDR levels of all four procedures are controlled at 0.1 approximately, and BH procedure is quite conservative. **b** The FNR yielded by oracle repLIS procedure, data-driven repLIS procedure and repfdr procedure are almost the same, and all of them are smaller than that of BH procedure. **c** The ATP yielded by oracle repLIS procedure, data-driven repLIS procedure and repfdr procedure are almost the same, and all of them are larger than that of BH procedure

In this scenario, we set *σ*_1_=*σ*_2_=1 and *μ*_2_=4. The joint states of the hypotheses across two studies $\left \{\left (H_{1,j},H_{2,j}\right)\right \}^{m}_{j=1}$ are generated from the Multinomial distribution *Multi*(10000,(0.4,0.2,0.2,0.2)). We vary *μ*_1_ from 2.0 to 3.0 with an increment 0.5 and exhibit the simulation results in Fig. 4. In Fig. 4, we can see from panel (a) that all four procedures can control the FDR level at the pre-specified level 0.1 approximately. Although the data-driven repLIS procedure has the largest FDR, it is still acceptable (FDR = 0.115). We can also observe that the empirical Bayes procedure repfdr is slightly conservative and the BH procedure leads to a quite small FDR value. These results indicate that our novel procedures are still valid for replicated analysis even the tests are independent in each study. The results revealed from panel (b) and (c) in Fig. 4 show that: (1) The FNR yielded by these procedures are decreasing when *μ*_1_ varies from 2.0 to 3.0; (2) The ATP yielded by these procedures are increasing when *μ*_1_ varies from 2.0 to 3.0; (3) The FNR and ATP yielded by oracle repLIS procedure, data-driven repLIS procedure, and repfdr procedure are almost the same. We can conclude that our proposed procedures (repLIS.or and repLIS) are as efficient as repfdr when the tests are independent in each study.

#### Scenario 2: locally dependent tests

**Fig. 5:**
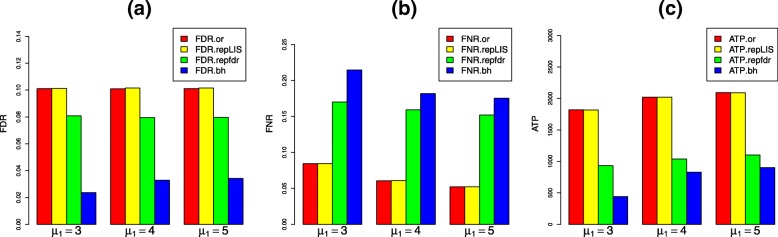
Simulation results in Scenario 2. **a** The FDR levels of all four procedures are controlled at 0.1, and the FDR yielded by oracle repLIS and data-driven are almost the same. **b** The FNR yielded by repfdr procedure and BH procedure are apparently large. **c** The ATP yielded by repfdr procedure and BH procedure are apparently small

In this scenario, we set *σ*_1_=*σ*_2_=1, *μ*_2_=2, and vary *μ*_1_ from 3 to 5 with an increment 1. Consider the CHMM (1)-(3) and the joint states of the hypotheses across two studies $\left \{\left (H_{1,j},H_{2,j}\right)\right \}^{m}_{j=1}$ are generated with the following transition matrix

$$A=\left(\begin{array}{cccc} 0.7 & 0.1 & 0.1 & 0.1\\ 0.1 & 0.7 & 0.1 & 0.1\\ 0.1 & 0.1 & 0.7 & 0.1\\ 0.1 & 0.1 & 0.8-A_{(1, 1)(1, 1)} & A_{(1, 1)(1, 1)}\\ \end{array} \right), $$

and the initial distribution *π* is set to be (0.25,0.25,0.25,0.25). Since the replicated associations are more likely to be clustered, the values of the entries in the diagonal of the transition matrix are set to be large. Here, *A*_(1,1)(1,1)_ is set to be 0.7, and the numerical results are displayed in Fig. 5. We further explored the robustness of repLIS under CHMMs by varying *A*_(1,1)(1,1)_ from 0.5 to 0.7, and the results are illustrated in Additional file 2.

To investigate the robustness of repLIS when the order of Markov dependence is incorrectly specified, we added simulation studies. Without loss of generality, we consider the case where the order of Markov dependence is set to be 2. We choose the setup to be consistent with those in Scenario 2 when possible. The detailed model settings are depicted in Additional file 2.

From Fig. 5 we can observe that the numerical results are almost coincide with those in Scenario 1, except that there is a significant difference in FNR and ATP values between our procedures (repLIS.or and repLIS) and repfdr procedure. The results reveal that our proposed procedures enjoy a smaller value of FNR and a larger value of ATP compared with their competitors. This indicates that our novel procedures are more efficient in detecting replicated signals when the tests are locally dependent in each study.

It is important to point out that the superiority of repLIS is achieved by characterizing the clustered and locally dependent structure via the Markov chain. Table 2 presents the outcomes of repLIS, repfdr, and BH in testing two clusters of replicated signals in Scenario 2 of Simulation I. It can be clearly seen that BH and repfdr can only identify the replicated signals with extremely small *p*-values, whereas repLIS tends to identify the entire cluster of replicated signals. By leveraging information from adjacent SNPs, repLIS are more efficient in detecting replicated signals.

**Table 2:** The significance levels suggested by BH, repfdr and repLIS

Sequence	States	Maximum	repfdr	repLIS	BH	repfdr	repLIS
		*p*-values	values	values	procedure	procedure	procedure
1027	∙	1.94e-1	5.48e-1	1.67e-1	∘	∘	∙
1028	∙	4.19e-3	4.59e-2	8.78e-3	∘	∙	∙
1029	∙	3.95e-2	2.28e-1	5.80e-2	∘	∙	∙
1030	∙	1.13e-1	3.79e-1	8.89e-2	∘	∘	∙
1031	∙	3.51e-3	2.88e-2	1.89e-2	∙	∙	∙
⋮	⋮	⋮	⋮	⋮	⋮	⋮	⋮
7305	∙	1.47e-3	2.21e-2	3.48e-3	∙	∙	∙
7306	∙	1.85e-2	2.16e-1	4.34e-2	∘	∙	∙
7307	∙	4.56e-2	2.07e-1	5.88e-2	∘	∙	∙
7308	∙	1.10e-1	3.73e-1	9.81e-2	∘	∘	∙
7309	∙	3.01e-2	3.35e-1	6.96e-2	∘	∘	∙
7310	∙	3.04e-4	8.18e-3	1.04e-2	∙	∙	∙

’ ∘’ denotes a null hypothesis or an acceptance and ’ ∙’ denotes a non-null hypothesis or a rejection. By exploiting the dependence information among adjacent SNPs, repLIS procedure tends to select disease-associated SNPs in clusters

### Ranking efficiency

**Fig. 6:**
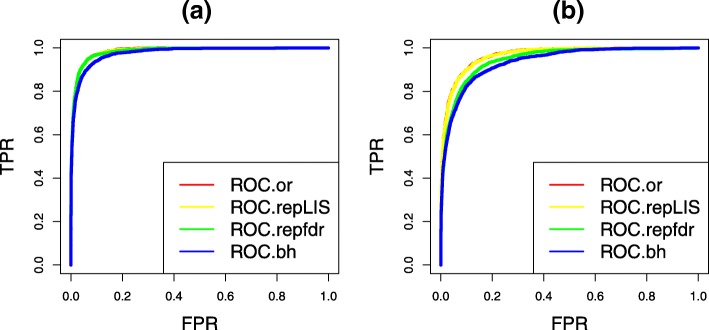
Comparisons of ranking efficiency. **a** The ROC curves under the model settings: ***μ***_***1***_=2.5,***μ***_***2***_=4,*σ*_***1***_=*σ*_***2***_=***1*** and the tests of all the SNPs are independent. **b** The ROC curves under the model settings: ***μ***_***1***_=3,***μ***_***2***_=2,*σ*_***1***_=*σ*_***2***_=***1*** and the tests of all the SNPs are under Markov dependence

The efficiency of ranking hypotheses is another measure that was widely used to perform comparison for different multiple testing procedures. In general, an efficient multiple testing procedure enjoys a ranked list where the non-nulls concentrate on the top of the ranked list. In this section, we use the ROC curve to compare the efficiency of ranking non-null hypotheses for different procedures. Figure 6 shows the results of the comparison for two cases that the tests of all the SNPs are independent (panel (a)) and are not independent (panel (b)) in each study, respectively. We can see that the ROC curves of our procedures dominate these of repfdr and BH procedures in panel (b). This implies that our repLIS procedures lead to a more efficient hypotheses ranking, especially when the tests of all the SNPs are not independent in each study.

### Simulation II

**Fig. 7:**
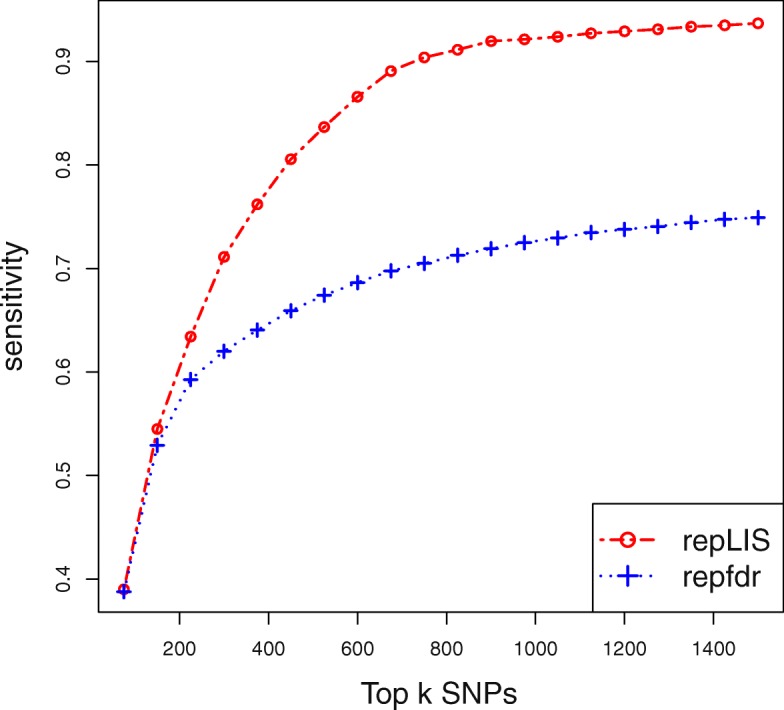
The sensitivity curves yielded by repLIS and repfdr in Simulation II. The three SNPs, 1200th, 1500th, 1800th, are chosen to be far away and the others, 6500th, 6504th, 6508th, are chosen to be clustered. The performance of replicability analysis procedure is assessed by the selection rate of relevant SNPs, which are defined as the three adjacent SNPs on each side of a causal SNP. The sensitivity is defined as the percentages of relevant SNPs that are selected by top *k* SNPs

In this section, we perform additional simulations to evaluate the performance of our repLIS procedure on a more realistic simulated data. In order to obtain a simulated data for two GWAS studies with more realistic LD patterns, we generate two genotype pools by randomly matching 340 haplotypes from the subjects of JPT+CHB (Japanese in Tokyo, Japan and Han Chinese in Beijing, China) and 410 haplotypes from the subjects of CEU+TSI (Utah residents with Northern and Western European ancestry from the CEPH collection and Toscani in Italia) collected by HapMap3 [35], respectively. To focus on the main points, we select six SNPs from a region of the chromosome 7 (consists of 10000 SNPs) as disease causal SNPs. Specifically, the three SNPs, 1200th, 1500th, 1800th, are chosen to be far away and the others, 6500th, 6504th, 6508th, are chosen to be clustered. The disease status *Y* is generated by using a logistic regression model:

$$\text{logit}(P(Y=1|G))=\beta_{0}+\sum\limits^{6}_{i=1}\beta_{i}G_{i}, $$

where *G*=(*G*_1_,*G*_2_,...,*G*_6_)^*T*^ and *G*_*i*_ is the corresponding genotype of the *i*th causal SNPs. We set *β*_0_=−8 and *β*_1_=*β*_2_=...=*β*_6_= log(2) so that the prevalence of the disease is controlled at 0.04. The performance of replicability analysis procedure is assessed by the selection rate of relevant SNPs, and the relevant SNPs are refered to as the three adjacent SNPs on each side of a causal SNP. The sensitivity is defined as the percentages of relevant SNPs that are selected by top *k* SNPs. The simulation is repeated for 100 times and the results are displayed in Fig. 7.

From Fig. 7 we can observe that the sensitivities yielded by our repLIS are uniformly larger than those of repfdr. This indicates that repLIS achieves a higher ranking efficiency and can discover more replicated signals at the same number of rejections.

## Additional file

Additional file 1 Brief description of some core code of our repLIS procedure. repLIS is a program to perform replicability analysis in genome-wide association studies, which is written in R code. Here, repLIS program is designed for one chromosome or a segment of chromosome. For the analysis of multiple chromosomes, firstly, the users can make the parallel computing for them, then complete the global analysis by combining all results from multiple chromosomes. (PDF 151 kb)

Additional file 2 Proof of Theorem 1 and additional simulations. We give a brief proof of Theorem 1 in Additional file 2. The asymptotic optimality can be derived without essential difficulty by extending the proof of Theorem 6 in [20]. We also carried out additional simulation studies to investigate the numerical performance of repLIS in various model settings. (PDF 249 kb)

## Abbreviations

GWAS: Genome-wide association studies
CHMM: Cartesian hidden Markov model
SNPs: Single nucleotide polymorphisms
BH: The Benjamini-Hochberg procedure
repLIS: The replicated local index of significance procedure
FDR: False discovery rate
FNR: False non-discovery rate
MCMC: Markov chain Monte Carlo algorithm
PGC: Psychiatric Genomics Consortium
WTCCC: Wellcome trust case control consortium
ADHD: Attention deficit-hyperactivity disorder
ASD: Autism spectrum disorder
BD: Bipolar disorder
MDD: Major depressive disorder
SCZ: Schizophrenia
