# Supplementary material for: Replicability analysis in genome-wide association studies via Cartesian hidden Markov models
Source: BMC Bioinformatics. 2019 Mar 18;20:146. doi: 10.1186/s12859-019-2707-7 (PMC6423849; doi:10.1186/s12859-019-2707-7)
Supplement: Supplementary file 1 — Brief description of some core code of our repLIS procedure. repLIS is a program to perform replicability analysis in genome-wide association studies, which is written in R code. Here, repLIS program is designed for one chromosome or a segment of chromosome. For the analysis of multiple chromosomes, firstly, the users can make the parallel computing for them, then complete the global analysis by combining all results from multiple chromosomes. (PDF 151 kb) [file 12859_2019_2707_MOESM1_ESM.pdf]

Welcome to repLIS - Replicability analysis in genome-wide association studies via Cartesian hidden Markov models

Pengfei Wang, and Wensheng Zhu*

August 21, 2018

Description

repLIS is a program to perform replicability analysis in genome-wide association studies, which is written in R code. Here, repLIS program is designed for one chromosome or a segment of chromosome. For the analysis of multiple chromosomes, firstly, the users can make the parallel computing for them, then complete the global analysis by combining all results from multiple chromosomes.

Citations

1. Sun W, Cai T. Large-scale multiple testing under dependence. Journal of the Royal Statistical Society: Series B (Statistical Methodology). 2009;71(2):393–424.
2. Wang P, Zhu W. Replicability analysis in genome-wide association studies via Cartesian hidden Markov models.

Downloads

- Some core code of repLIS procedure are available on GitHub (https://github.com/wpf19890429/large-scale-multiple-testing-via-CHMM).
  This repository contains the following files:
  rdata.hmm.Cartesian.R
  bwfw.hmm.Cartesian.R
  em.hmm.Cartesian.R
  bwfw.hmm3.Cartesian.R
  em.hmm3.Cartesian.R
  mt.hmm.R.txt

illustrations of core R functions

1. rdata.hmm.Cartesian

Description:

Generating the observed z-valuse and the states of hypotheses that are based on Cartesian hidden Markov models.

Usage:

rdata.hmm.Cartesian(NUM, pii, A, f0, f1, f2)

Arguements:

NUM: sample size

pii=(pii[1], pii[2],pii[3],pii[4]): initial state distribution

A: transition matrix

f0: parameter set for the null distribution

f1: parameter set of the non-null distribution for study 1

f2: parameter set of the non-null distribution for study 2

Values:

x1: continuous observed z-values from study 1

x2: continuous observed z-values from study 2

theta: four-state unobserved states of hypotheses

2. bwfw.hmm.Cartesian

Description:

Calculating the repLIS multiple testing statistics via the backward-forward algorithm.

Usage:

bwfw.hmm.Cartesian(x1, x2, pii, A, f0, f1, f2)

Arguements:

x1: continuous observed z-values from study 1

x2: continuous observed z-values from study 2

pii=(pii[1], pii[2],pii[3],pii[4]): initial state distribution

A: transition matrix

f0: parameter set of the null distribution

f1: parameter set of the non-null distribution for study 1

f2: parameter set of the non-null distribution for study 2

Values:

alpha: rescaled backward variables

beta: rescaled forward variables

lfdr: repLIS multiple testing statistics

3. em.hmm.Cartesian

Description:

Calculating the parameters of Cartesian hidden Markov models via the EM algrithm.

Usage:

em.hmm.Cartesian(x1, x2, maxiter=200)

Arguements:

x1: continuous observed z-values from study 1

x2: continuous observed z-values from study 2

maxiter: the maximum number of iterations

Values:

pii.new: the estimation of initial state distribution

A.new: the estimation of transition matrix

f1.new: the estimation of parameter set for the non-null distribution for study 1

f2.new: the estimation of parameter set for the non-null distribution for study 2

niter: the number of iterations

4. bwfw.hmm3.Cartesian

Description:

Calculating the repLIS multiple testing statistics for multiple (=3) GWAS studies via the backward-forward algorithm.

Usage:

bwfw.hmm3.Cartesian(x1, x2, x3, pii, A, f0, f1, f2, f3)

Arguements:

x1: continuous observed z-values from study 1

x2: continuous observed z-values from study 2

x3: continuous observed z-values from study 3

pii=(pii[1], pii[2],pii[3],pii[4],pii[5],pii[6],pii[7],pii[8]): initial state distribution

A: transition matrix

f0: parameter set of the null distribution

f1: parameter set of the non-null distribution for study 1

f2: parameter set of the non-null distribution for study 2

f3: parameter set of the non-null distribution for study 3

Values:

alpha: rescaled backward variables

beta: rescaled forward variables

lfdr: repLIS multiple testing statistics

5. em.hmm3.Cartesian

Description:

Calculating the parameters of Cartesian hidden Markov models for multiple (=3) GWAS studies via the EM algrithm.

Usage:

em.hmm.Cartesian(x1, x2, x3, maxiter=200)

Arguements:

x1: continuous observed z-values from study 1

x2: continuous observed z-values from study 2

x3: continuous observed z-values from study3

maxiter: the maximum number of iterations

Values:

pii.new: the estimation of initial state distribution

A.new: the estimation of transition matrix

f1.new: the estimation of parameter set for the non-null distribution for study 1

f2.new: the estimation of parameter set for the non-null distribution for study 2

f3.new: the estimation of parameter set for the non-null distribution for study 3

niter: the number of iterations

6. mt.hmm

Description:

Conducting repLIS procedure when a pre-specified nominal level is given.

Usage:

mt.hmm(repLIS, q)

Arguements:

repLIS: repLIS multiple testing statistics

q: the pre-specified nominal level

Values:

nr: the number of rejected hypotheses

th: the threshold

re: the rejected hypotheses

ac: the accepted hypotheses

de: the decision rule

Examples:

```
## the number of observed z-values
source("rdata.hmm.Cartesian.R")
NUM<-10000
## initialize the transition matrx
A<-matrix(c(c(0.7, 0.1, 0.1, 0.1),
            c(0.1, 0.7, 0.1, 0.1),
            c(0.1, 0.1, 0.7, 0.1),
            c(0.1, 0.1, 0.1, 0.7)), 4, 4, byrow=TRUE)

## initialize parameter set of the null and non-null distributions
f0<-c(0, 1)
f1<-c(1, 1)
f2<-c(3, 1)

## initialize state distribution
pii<-c(0.25, 0.25, 0.25, 0.25)


## Generating the observed z-values and the states of hypotheses that are based on
## Cartesian hidden Markov models.
rdata<-rdata.hmm.Cartesian(NUM, pii, A, f0, f1, f2)
x1<-rdata$x1
x2<-rdata$x2
theta<-rdata$s

## The input file consists of the above generated z-values
Simulation_data<-read.table("input_data.csv",header=TRUE,sep=",")
x1<- Simulation_data[, 2]
x2<- Simulation_data[, 3]
theta<- Simulation_data[, 4]
```

```
## Calculating the repLIS multiple testing statistics via the backward-forward
## algorithm.
source("rdata.hmm.Cartesian.R")
source("bwfw.hmm.Cartesian.R")

bwfw.res<-bwfw.hmm.Cartesian(x1, x2, pii, A, f0, f1, f2)
repLIS<-bwfw.res$lsi

## Conducting repLIS procedure given the pre-specified level is 0.1.
res.repLIS<-mt.hmm(repLIS,0.1)$de
res.theta<-rep(0, 10000)
res.theta[theta==4]<-1
N10<-length(which(res.repLIS-res.theta>0))
R<-length(which(res.repLIS==1))+0.0001
FDR<-N10/R

## The output file consists of the simulation results of the replicated signals (=1)
output<- res.theta
colname<- " res.theta "
colnames(output)<-colname
file_name<-paste("Result",".csv",sep="")
write.csv(output, file=file_name)

## In real data analysis, the parameters of Cartesian hidden Markov models are unknown.
## Firstly, we need to conduct the EM algorithm for estimating the parameters of CHMM.
## x1: continuous observed z-values from study 1
## x2: continuous observed z-values from study 2
em<-em.hmm.Cartesian(x1, x2, 100)
em.res<-bwfw.hmm.Cartesian(x1, x2, em$pii, em$A, f0, em$f1, em$f2)
```
