# Supplementary material for: Replicability analysis in genome-wide association studies via Cartesian hidden Markov models
Source: BMC Bioinformatics. 2019 Mar 18;20:146. doi: 10.1186/s12859-019-2707-7 (PMC6423849; doi:10.1186/s12859-019-2707-7)
Supplement: Supplementary file 2 — Proof of Theorem 1 and additional simulations. We give a brief proof of Theorem 1 in Additional file 2. The asymptotic optimality can be derived without essential difficulty by extending the proof of Theorem 6 in [20]. We also carried out additional simulation studies to investigate the numerical performance of repLIS in various model settings. (PDF 249 kb) [file 12859_2019_2707_MOESM2_ESM.pdf]

# Supplement: Replicability analysis in genome-wide association studies via Cartesian hidden Markov models [1]

## 1. Proof of Theorem 1

*Proof.* Let $\delta(repLIS, c_\alpha) = \{\delta_j = I_{(repLIS_j < c_\alpha)}, j = 1, ..., m,\}$ be the classification rule corresponding to the repLIS procedure (5). The FDR level of the repLIS procedure is

$$\begin{aligned} FDR_{repLIS} &= E\left\{\frac{\sum_j[(1-H_{1,j})(1-H_{2,j})+H_{1,j}(1-H_{2,j})+(1-H_{1,j})H_{2,j}]\delta_j}{(\sum_j \delta_j)\vee 1}\right\} \\ &= E\left\{\frac{1}{(\sum_j \delta_j)\vee 1}[\sum_j \delta_j repLIS_j]\right\}. \end{aligned}$$

Note that $\sum_j \delta_j repLIS_j \leq \alpha \sum_j \delta_j$, we have $FDR_{repLIS} \leq E\left\{\frac{1}{(\sum_j \delta_j)\vee 1}\alpha(\sum_j \delta_j)\right\} \leq \alpha$. The asymptotic optimality can be derived without essential difficulty by extending the proof of Theorem 6 in Sun and Cai (2009). □

## 2. Additional Simulation Results

In this section, we carried out additional simulation studies to investigate the numerical performance of repLIS in various model settings. Here, it is necessary to note that the repLIS's competitor, repfdr, is carried out in an ideal case that the proportions of each joint hypotheses states are known. In practice, however, these proportions are usually unknown and repfdr can be more conservative.

[1] This work is supported in part by the Natural Science Foundation of China Grants 11771072 and 11371083.

## 2.1. More stringent FDR level

The model settings are coincide with those in Scenario 2 of Simulation I. Since the BH procedure with FDR level 0.02 is too conservative to identify replicated signals, we removed BH procedure from simulation studies. Figure 1 contains the simulation results with more stringent FDR level (0.02). We can observe that the FDR levels of all three procedures are controlled at 0.02 approximately and both oracle and data-driven repLIS dominate repfdr when $\mu_1$ varies from 3 to 5.

## 2.2. The robustness of repLIS when $A_{(1,1)(1,1)}$ varies

In this section, we explored the robustness of repLIS under CHMMs, when the component of transition probability was varied. In this scenario, we set $\sigma_1 = \sigma_2 = 1$, $\mu_1 = 3$ and $\mu_2 = 2$. The joint states $\{(H_{1,j}, H_{2,j})\}_{j=1}^{m}$ are generated with the following transition matrix:

$$A = \begin{pmatrix} 0.7 & 0.1 & 0.1 & 0.1 \\ 0.1 & 0.7 & 0.1 & 0.1 \\ 0.1 & 0.1 & 0.7 & 0.1 \\ 0.1 & 0.1 & 0.8 - A_{(1,1)(1,1)} & A_{(1,1)(1,1)} \end{pmatrix},$$

and the initial distribution $\pi$ is set to be $(0.25, 0.25, 0.25, 0.25)$. We varied $A_{(1,1)(1,1)}$ from 0.5 to 0.7 with an increment 0.05. The numerical results are displayed in Figure 2. Note that the larger value of $A_{(1,1)(1,1)}$, the higher cluster level of replicated signals. It is easy to interpret that the larger value of $A_{(1,1)(1,1)}$, the larger value of ATP yielded by repLIS. We can also concluded from Figure 2 that the performance of the oracle repLIS can be attained by the data-driven repLIS asymptotically and both oracle and data-driven repLIS uniformly outperform the repfdr in finding replicated signals.

## 2.3. The robustness of repLIS when the order of Markov dependence is incorrectly specified

Without loss of generality, we consider the case where the order of Markov dependence is set to be 2. We chose the setup to be consistent with those in Scenario 2 of Simulation I when possible. Specifically, we set $\sigma_1 = \sigma_2 = 1$ and $\mu_2 = 2$. For simplify, consider the following transition matrix:

$$\{A^{(2)}_{i,j}\}_{4\times 4} = \begin{pmatrix} 0.7 & 0.1 & 0.1 & 0.1 \\ 0.1 & 0.7 & 0.1 & 0.1 \\ 0.1 & 0.1 & 0.7 & 0.1 \\ 0.1 & 0.1 & 0.1 & 0.7 \end{pmatrix},$$

where $A^{(2)}_{uv} = P\left((H_{1,j+2}, H_{2,j+2}) = v|(H_{1,j}, H_{2,j}) = u\right)$, for $u, v \in \{(0,0),(1,0),(0,1),(1,1)\}$ and $j = 1, ..., m-1$. We varied $\mu_1$ from 3 to 5 with an increment 1. The simulation results are depicted in Figure 3. the oracle repLIS is implemented by using $\{A^{(2)}_{i,j}\}_{4\times 4}$ to replace the corresponding $\{A_{i,j}\}_{4\times 4}$. It is easy to see that the performance of the data-driven repLIS is still acceptable (FDR=0.115). This implies that the data-driven repLIS can adaptively adjust for parameter estimations when the order of Markov dependence is incorrectly specified. Here, the superiority of repLIS is achieved by using the information that the proportions of each joint hypotheses states are known.

## 2.4. Extend repLIS to multiple GWAS studies

To focus on the main ideas, we restrict attention to repLIS in testing two GWAS studies. Extending repLIS to the general multiple ($>= 3$) GWAS studies is formally straightforward, but requires additional computation. In this section, we carried out further simulation studies for repLIS in testing three GWAS studies. We set $\sigma_1 = \sigma_2 = \sigma_3 = 1$ and $\mu_2 = \mu_3 = 2$. The triple states $\{(H_{1,j}, H_{2,j}, H_{3,j})\}_{j=1}^{m}$ are generated with the following transition

matrix:

$$\{B_{i,j}\}_{8\times 8} = \begin{pmatrix} 0.65 & 0.05 & 0.05 & 0.05 & 0.05 & 0.05 & 0.05 & 0.05 \\ 0.05 & 0.65 & 0.05 & 0.05 & 0.05 & 0.05 & 0.05 & 0.05 \\ 0.05 & 0.05 & 0.65 & 0.05 & 0.05 & 0.05 & 0.05 & 0.05 \\ 0.05 & 0.05 & 0.05 & 0.65 & 0.05 & 0.05 & 0.05 & 0.05 \\ 0.05 & 0.05 & 0.05 & 0.05 & 0.65 & 0.05 & 0.05 & 0.05 \\ 0.05 & 0.05 & 0.05 & 0.05 & 0.05 & 0.65 & 0.05 & 0.05 \\ 0.05 & 0.05 & 0.05 & 0.05 & 0.05 & 0.05 & 0.65 & 0.05 \\ 0.05 & 0.05 & 0.05 & 0.05 & 0.05 & 0.05 & 0.05 & 0.65 \end{pmatrix},$$

and the initial distribution $\pi$ is set to be $(0.125, 0.125, 0.125, 0.125, 0.125, 0.125, 0.125, 0.125)$. We varied $\mu_1$ from 2 to 3 with an increment 0.5 and the detailed simulation results are displayed in Figure 4.

We can observe that the simulation results for three GWAS studies are almost coincide with those for testing two GWAS studies. We can also validate the robustness of repLIS when the transition matrix is modified or the order of Markov dependence is incorrectly specified, if desired. The validation of the robustness is standard as described in Section 2.2 and 2.3, so we won't reiterate it here.

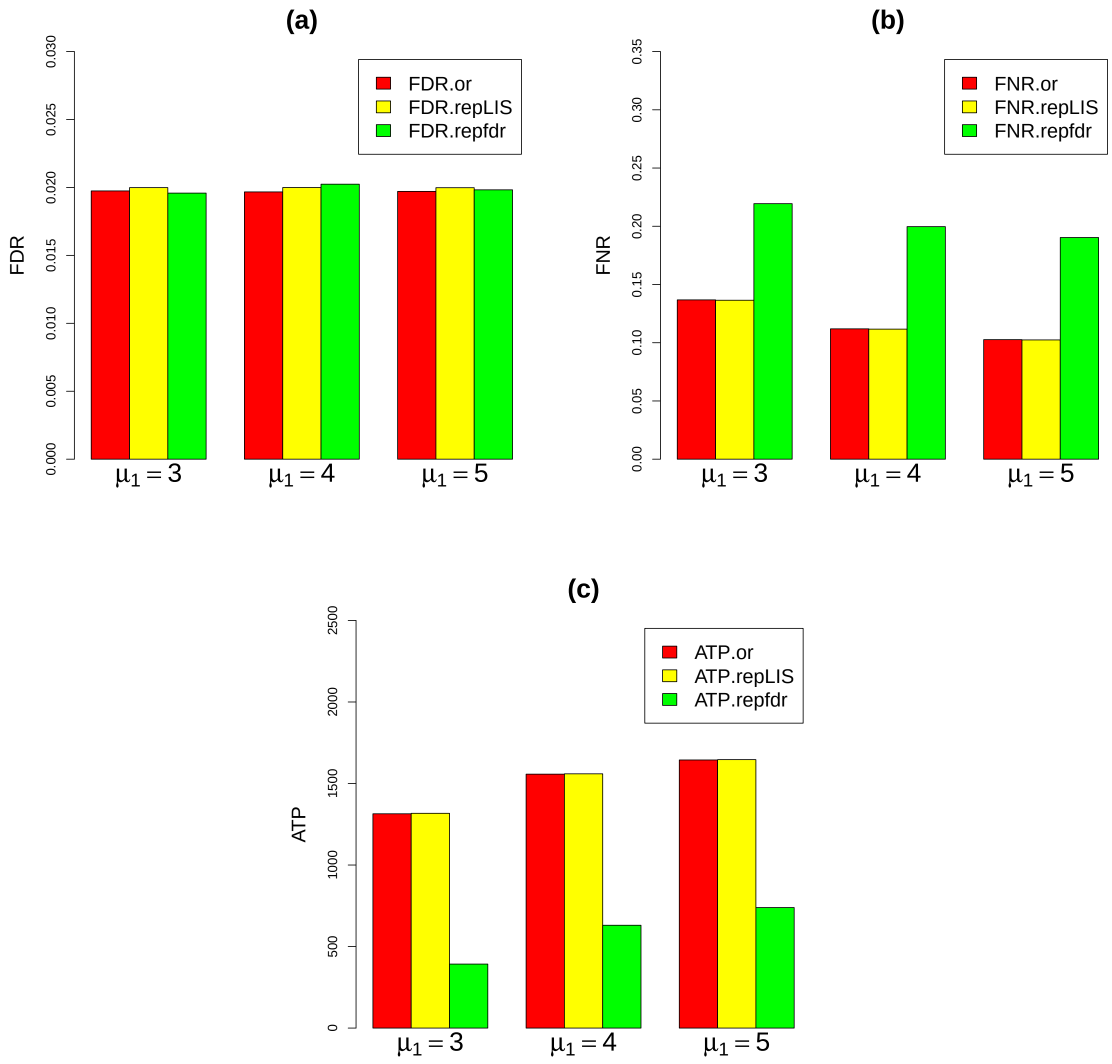


Figure 1: **Simulation results in Scenario 2 with FDR=0.02**. (a) The FDR levels of all three procedures are controlled at 0.02 approximately. (b) Both oracle and data-driven repLIS dominate repfdr when $\mu_1$ varies from 3 to 5.

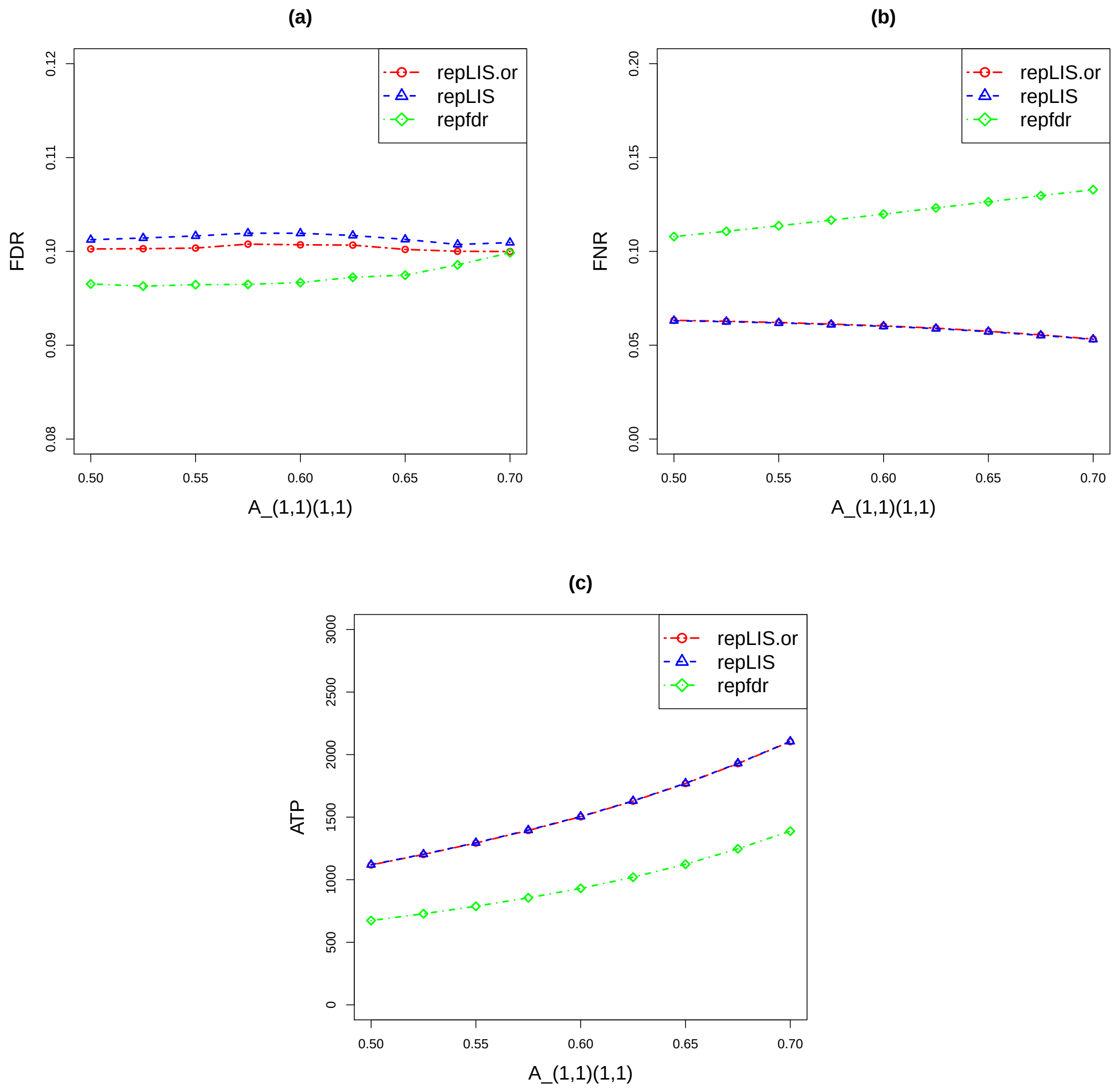


Figure 2: **Simulation results when $A_{(1,1)(1,1)}$ varies.** (a) The performance of the oracle repLIS (repLIS.or) can be attained by the data-driven repLIS (repLIS) asymptotically. (b) Both oracle and data-driven repLIS uniformly outperform the repfdr in finding replicated signals.

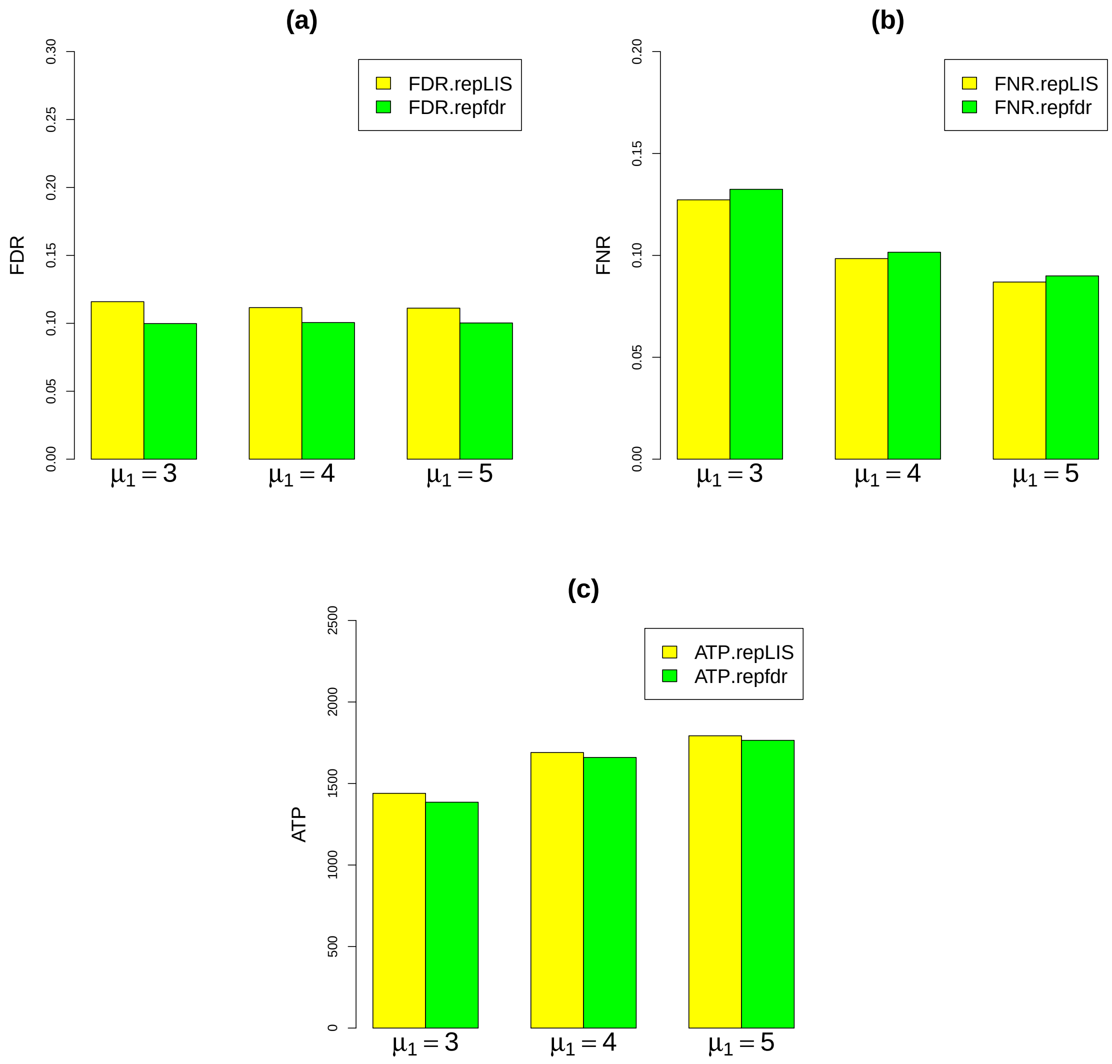


Figure 3: **Simulation results in Scenario 2 with misspecified Markov dependence**. The performance of the data-driven repLIS is still acceptable (FDR=0.115).

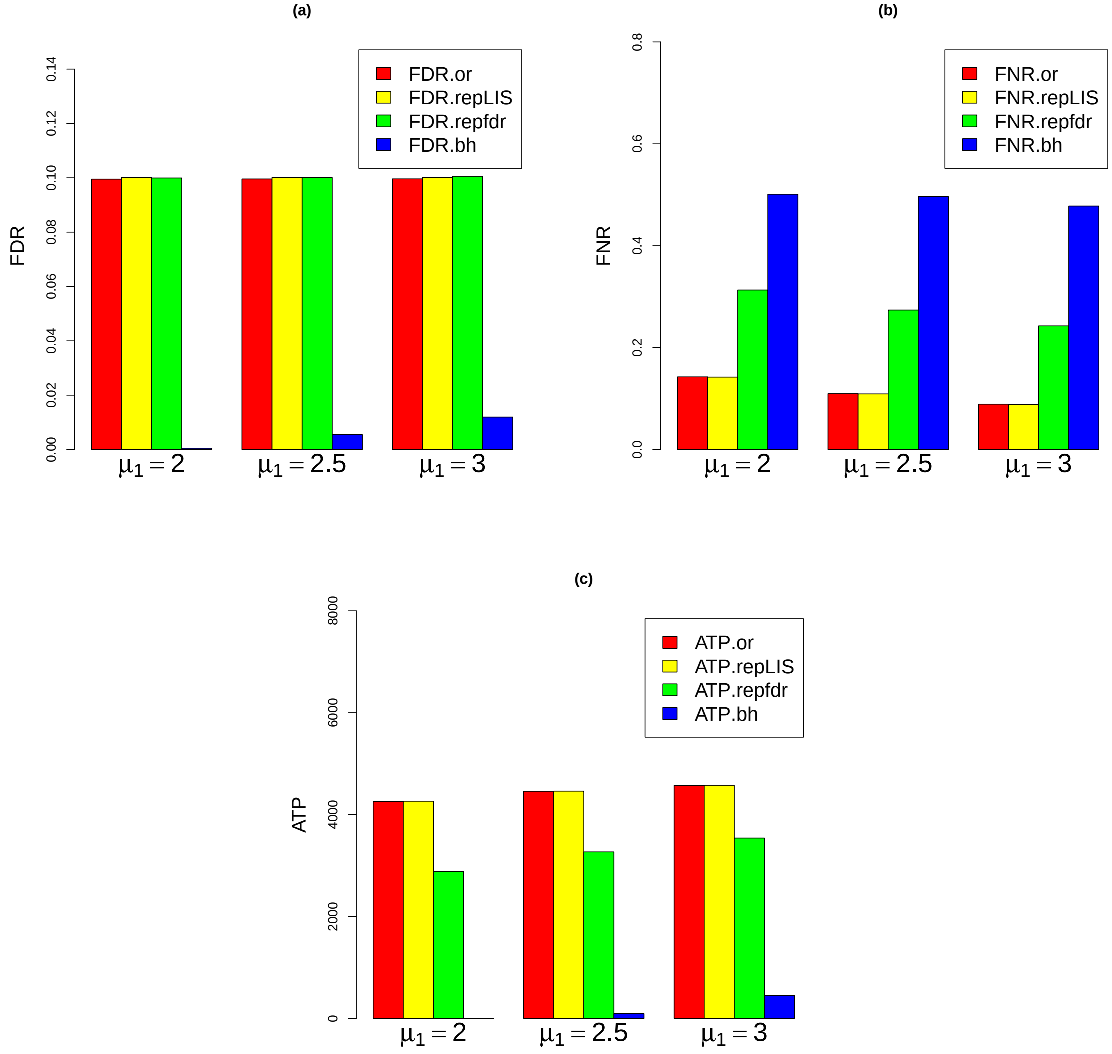


Figure 4: **Simulation results for three GWAS studies**. The numerical results are almost coincide with those for testing two GWAS studies.
